# Molecular mechanisms of human IRE1 activation through dimerization and ligand binding

**DOI:** 10.18632/oncotarget.3864

**Published:** 2015-04-18

**Authors:** Amar Joshi, Yvette Newbatt, P. Craig McAndrew, Mark Stubbs, Rosemary Burke, Mark W. Richards, Chitra Bhatia, John J. Caldwell, Tatiana McHardy, Ian Collins, Richard Bayliss

**Affiliations:** ^1^ Department of Biochemistry, University of Leicester, Leicester, United Kingdom; ^2^ Cancer Research UK Leicester Centre, University of Leicester, Leicester, United Kingdom; ^3^ Cancer Research UK Cancer Therapeutics Unit, Division of Cancer Therapeutics, The Institute of Cancer Research, London, United Kingdom

**Keywords:** UPR, drug discovery, kinase, RNase

## Abstract

IRE1 transduces the unfolded protein response by splicing XBP1 through its C-terminal cytoplasmic kinase-RNase region. IRE1 autophosphorylation is coupled to RNase activity through formation of a back-to-back dimer, although the conservation of the underlying molecular mechanism is not clear from existing structures. We have crystallized human IRE1 in a back-to-back conformation only previously seen for the yeast homologue. In our structure the kinase domain appears primed for catalysis but the RNase domains are disengaged. Structure-function analysis reveals that IRE1 is autoinhibited through a Tyr-down mechanism related to that found in the unrelated Ser/Thr protein kinase Nek7. We have developed a compound that potently inhibits human IRE1 kinase activity while stimulating XBP1 splicing. A crystal structure of the inhibitor bound to IRE1 shows an increased ordering of the kinase activation loop. The structures of hIRE in apo and ligand-bound forms are consistent with a previously proposed model of IRE1 regulation in which formation of a back-to-back dimer coupled to adoption of a kinase-active conformation drive RNase activation. The structures provide opportunities for structure-guided design of IRE1 inhibitors.

## INTRODUCTION

The endoplasmic reticulum is responsible for folding secretory proteins. At homeostasis the folding capacity of the ER and the amount of secretory protein synthesis are balanced. However, if protein synthesis eclipses folding capacity, the accumulation of misfolded protein is sensed and a recovery mechanism, termed the unfolded protein response (UPR), is activated. Initially, UPR signaling reduces protein translation and increases the ER lumen volume and protein folding capacity, however if homeostasis cannot be restored the UPR signals for the cell to undergo apoptosis [1, 2].

The most evolutionarily conserved UPR pathway is the IRE1-bZIP pathway [3]. IRE1 resides in the ER membrane. It has an N-terminal stress sensing domain in the ER lumen and a cytoplasmic kinase/ribonuclease domain which are connected by a single-pass transmembrane helix [4]. The lumenal domains dimerise in response to ER stress bringing the cytoplasmic domains together [5]. IRE1 then undergoes autophosphorylation, generating a specific endoribonuclease activity that hydrolyses 2 stem-loops in the pre-mRNA for a bZIP transcription factor [5, 6]. Re-ligation of the RNA results in a frame shift, and subsequently an active transcription factor is translated, HAC1 in yeast and XBP1 in metazoans [7], which increases transcription of UPR target genes [1, 2].

Recent evidence has linked the UPR to numerous diseases. Specifically, the pro-survival IRE1-XBP1 pathway has a role in disease progression for inflammatory bowel diseases, metabolic disorders and cancers [8-11]. The requirement for the IRE1-XBP1 pathway in myeloma and the recent discovery of a key function for this pathway in the progression of aggressive, triple-negative breast cancer has meant that IRE1 has become the focus of several drug discovery programs [12-16]. A detailed understanding of the IRE1 activation mechanism in humans will accelerate therapeutic development.

The regulation of IRE1 RNase activity is unusual and not completely understood, but key parameters are known. RNase activity is coupled to autophosphorylation of the kinase domain activation loop, which is separated from the RNase active site by approximately 50 Å [17, 18]. Although it might be expected that ATP-competitive inhibitors that inhibit autophosphorylation would also block RNase activity, a subset of inhibitors stimulate RNase activity [19], perhaps by stabilizing a conformation that mimics phosphorylated IRE1. The structure of phosphorylated yeast IRE1 (yIRE1), which represents a state of high RNase activity, revealed a dimer in a back-to-back conformation [20]. The kinase N-lobe and RNase domains form extensive contacts in the dimeric interface and a continuous surface is formed by the united RNase domains. Within the kinase domain, the active-site resembles a canonical kinase active site and the activation loop is stabilized [18, 20, 21]. By contrast the structure of the dephosphorylated human IRE1 (hIRE1)-ADP complex, which represents a state of low RNase activity, shows hIRE1 protomers in a face-to-face orientation in which the RNase domains are widely separated [22]. This orientation of the kinase domains directs the activation loop of one IRE1 molecule towards the active site of the facing IRE1 molecules, and thus provides a rationale for the mechanism of reciprocal autophosphorylation in trans. However, the kinase active site is lacking several hallmarks of an active kinase, as is often the case in structures of kinases determined without the activating phosphorylation [23]. Recently published structures of murine IRE1 (mIRE1) in complex with ADP and RNase domain inhibitors also exhibit face-to-face dimer conformations formed from protomers that, similar to hIRE1 bound to ADP, are in kinase-inactive conformations [24]. The conformation adopted by hIRE1 and mIRE1 ADP complexes is incompatible with back-to-back dimer formation [24, 25]. The structure of hIRE1 bound to an ATP-competitive inhibitor of kinase and RNase activity forms neither back-to-back dimers nor face-to-face dimers, suggesting perhaps that the compounds might stabilize a monomeric form of IRE1 [26].

These important structures underpin the current model of IRE1 RNase activation, in which trans-autophosphorylation between face-to-face IRE1 dimers generates an active RNase through back-to-back dimerization and higher-order multimerisation [18, 20, 25]. However, because the existing structures of the yeast and human/murine proteins exhibit very different overall conformations generated by dimerization through distinct interfaces, and because there are differences in their primary sequences at these interfaces, the extent to which the mechanisms of regulation are conserved is not completely clear. For example, although there is biochemical evidence in support of a back-to-back dimer of murine IRE1 [24], it is not known how closely it resembles the yeast structure. In this current work we present the structure of dephosphorylated human IRE1 in apo form and as a kinase inhibitor-bound complex. In these structures, hIRE1 forms a back-to-back dimer in which the protomers are twisted relative to their relative conformations in structures of yIRE1.

## RESULTS

### Crystal structure of apo-hIRE1 as a back-to-back dimer

**Figure 1:**
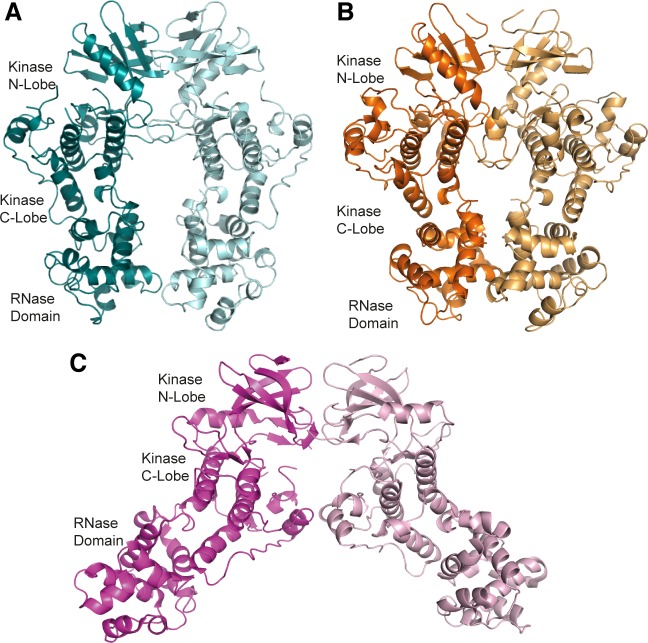
apo-hIRE1 forms a back-to-back dimer **A.** The back-to-back dimer conformation of apo-hIRE. Cartoon representation of the two chains of apo-hIRE1 in the crystal structure colored teal and pale blue respectively. **B.** Cartoon representation of the active phosphorylated yIRE1 dimer in a back-to-back orientation (PDB ID 3FBV) [18], individual chains are shown in light and dark orange. **C.** Cartoon representation of ADP-hIRE1 (PDB ID 3P23) [22], individual chains are shown in magenta and pink.

**Figure 2:**
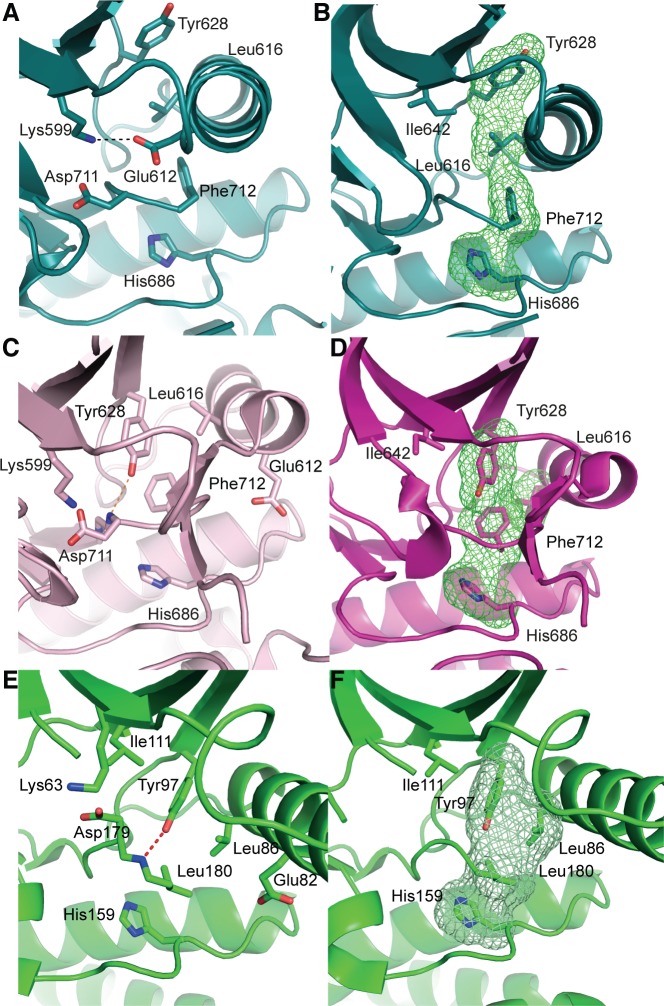
apo-hIRE1 is in a kinase pre-active conformation, whereas ADP-hIRE1 is in an autoinhibited conformation **A.** The kinase active site of apo-hIRE1. Main chain atoms shown in cartoon representation with selected side chain atoms shown. Atoms are colored by element: carbon, teal; oxygen, red; nitrogen, blue. The salt bridge between Lys599 and Glu612 is shown as a dashed black line. **B.** Aligned hydrophobic R-spine residues in the apo-hIRE1 N-lobe. The surface formed by the side chains of R-spine residues is shown as a green mesh. **C.** The kinase active site of ADP-hIRE1 crystal structure (PDB ID 3P23 [22]), shown in the equivalent view to A. Carbon atoms are colored pink. The hydrogen bonds between Tyr628 on β4 and Asp711 in the DFG motif is shown as a dashed orange lines. **D.** Non-aligned hydrophobic R-spine residues in the ADP-hIRE1 N-lobe, shown in the view equivalent to B. The surface formed by the side chains of R-spine residues is shown as a green mesh. **E.** The kinase active site of Nek7 is shown in cartoon representation with selected side chain and main chain atoms shown (equivalent view to A). The hydrogen bond between the Tyr97 side chain hydroxyl and Leu180 main chain amide is shown as a dashed red line. Main chain atoms are shown in cartoon representation. **F.** The active site of Nek7 (PDB ID 2WQM) [27] is in an autoinhibitory conformation. The surface formed by the side chains of R-spine residues is shown as a green mesh.

**Figure 3:**
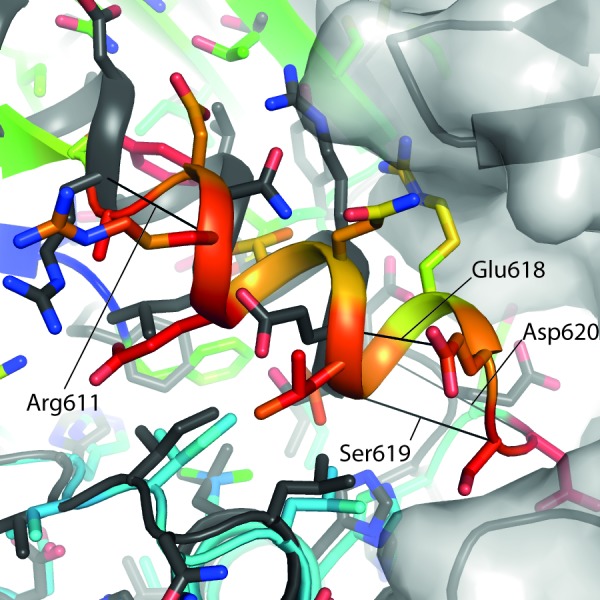
The αC-helix in the ADP-hIRE1 structure is incompatible with back-to-back dimer formation Cartoon representation of the apo-hIRE1 structure (grey), with a semitransparent grey surface shown for one monomer. Cartoon representation of a monomer from the ADP-IRE1 structure [22]. Carbon atoms are colored by RMSD from the alignment to the apo-IRE1 monomer (from blue – low to red – high), while oxygen atoms are colored red and nitrogen atoms colored blue. For selected residues a black line is drawn between Cα atoms from identical residues between the apo- and ADP-hIRE1 crystal structures.

The cytoplasmic region of human IRE1 (residues 547-977; hIRE1) was expressed in *Spodoptera frugiperda* (Sf9) cells. Purified, dephosphorylated hIRE1 (Figure S1A) crystallized in the absence of nucleotide and diffraction data were collected to 2.6 Å (Table 1). Our crystal structure shows apo-hIRE1 is a symmetrical dimer in a back-to-back conformation (Figure 1A) similar to the structure of phosphorylated yIRE1 (Figure 1B), and distinct from the face-to-face dimer previously observed in ADP-bound hIRE (Figure 1C). While the unphosphorylated activation loop is not observed in our structure, the kinase active site has features associated with a functional kinase: the conserved Lys-Glu salt bridge between Lys599 in β3 and Glu612 in αC is formed (Figure 2A); the side chains of Tyr628, Leu616, Phe712 and His686 form a continuous hydrophobic R-spine, although the side chain of Phe712 is mis-aligned (Figure 2B); and the gatekeeper Ile642 packs against the αC-helix [23]. This contrasts with the hIRE1-ADP structure determined previously that has a markedly different kinase active site in which the side chain of Tyr628 from β4 points down into the active site, forming hydrogen bonds to the DFG motif (Figure 2C) [22]. The intrusion of the aromatic side chain into the active site physically separates the gatekeeper Ile642 from the αC-helix. As previously noted [24], this rearrangement breaks the R-spine; Leu616 and Tyr628 are side-by-side rather than forming the continuous hydrophobic spine (Figure 2D). Furthermore the Lys-Glu salt bridge cannot form as the αC-helix is translated along its axis which, in combination with unwinding by a full helical turn, displaces equivalent Cα atoms by ≈7.5 Å between the two structures (Figure 3). The kinase active site of the hIRE1-ADP complex is not in a functionally active conformation, and the structure is similar to the autoinhibitory conformation that was first observed in the mitotic Ser/Thr kinase Nek7 where the equivalent tyrosine on β4 forms a hydrogen bond to a peptide amine within the DLG motif (Figure 2E, 2F) [27, 28].

**Table 1:** Crystal diffraction data and structure refinement statistics

Data collection	Apo-hIRE1	Imidazopyridine-hIRE1
Space-group	P 1 2_1_ 1	P 2_1_2_1_2_1_
Cell dimensions		
a, b, c (Å)	79.40, 78.97, 86.04	78.68,81.96,168.68
α, β, γ (°)	90.00, 97.41, 90.00	90.00,90.00,90.00
Wavelength (Å)	0.92	0.92
Resolution range (Å)	85.33-2.60 (2.72-2.60)	46.36-2.90 (3.08-2.90)
R_merge_	0.083 (1.475)	0.201 (1.44)
I/σI	7.8 (0.8)	7.5 (1.4)
CC_1/2_	0.996 (0.487)	0.944 (0.672)
Redundancy	3.4 (3.5)	6.6 (6.4)
Completeness (%)	99.6 (99.5)	99.7 (99.0)
		
**Refinement**		
Resolution (Å)	85.33 – 2.60	46.36-2.90
No. reflections	32479	24770
R_work_/R_free_	0.203/0.225	0.200/0.225
No. molecules in a.s.u.	2	2
No. atoms		
Protein	6025	6186
Water	15	21
B-factors		
Protein	101.2	87.0
Water	63.0	44.8
Ligands	56.6	75.4
RMS deviations		
Bond lengths (Å)	0.009	0.01
Bond angles (°)	1.13	1.14
Ramachandran plot (% favoured / allowed / outliers)	93.83/5.35/0.82	91.57/8.04/0.40
PDB Identifier	4Z7G	4Z7H

### Mutation of IRE1 Y628 accelerates autophosphorylation

**Figure 4:**
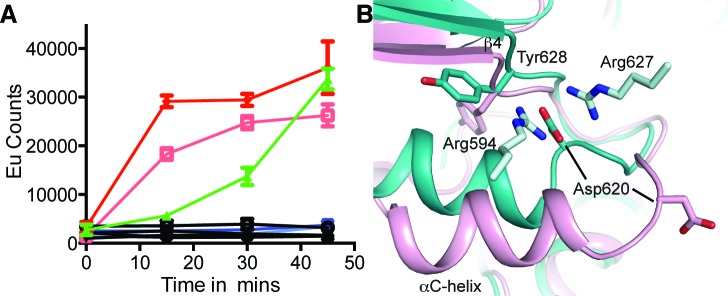
Mutation of hIRE1 Y628 enhances autophosphorylation **A.** Autophosphorylation of hIRE1 wild-type (wt) and Y628 mutants measured by DELFIA assay. Results are color-coded by protein variant, protein concentration and presence or absence of ATP at 100 μM ATP. Black lines - absence of ATP. Green - wt IRE1 at 700 nM. Dark and light red – IRE1 Tyr628Phe at 1400 nM and 700 nM, respectively. Dark blue and light blue – Tyr628Leu at 1400 nM and 700 nM, respectively. **B.** View of the conformational changes between the ADP-hIRE1 structure (pink carbon atoms) and the apo-hIRE1 structure in the vicinity of Tyr628. Note that Asp620 in the αC-β4 linker moves by ~8 Å upon back-to-back dimer formation, and forms salt-bridge interactions with Arg594 and Arg627 from a second molecule of hIRE1 in the back-to-back dimer interface.

In Nek7 a Tyr residue on β4 is involved in regulating auto-activation and mutation of this residue to phenylalanine, leucine or alanine stimulated kinase activity [27]. To examine whether Tyr628 has a similar function in the activation mechanism of IRE1 we generated mutations of Tyr628: Tyr628Phe and Tyr628Leu were designed to test the requirement for the hydrogen bond while maintaining the physicochemical properties of the amino acid while the equivalent of the Tyr628Ala mutation in Nek7 had the greatest activity. Mutations of IRE1 Tyr628 to alanine or leucine destabilized the protein and it could no longer auto-phosphorylate illustrating that IRE1 is more sensitive to amino acid perturbations at this position than Nek7. However, Tyr628Phe was stable and had greater autophosphorylation activity than wild-type IRE1 (Figure 4A) [29]. This result supports our interpretation of the hIRE1-ADP structure as representing an autoinhibited, inactive state of the protein. The hydrogen bonds that stabilize Tyr-down cannot be formed by the phenylalanine side chain in the Tyr628Phe mutant, and the autoinhibitory conformation is destabilized, leading to an enhanced rate of autoactivation. The physiological activation mechanism of IRE1 must involve a rearrangement of the active site that moves the side chain of Tyr628 from its buried position as found in the hIRE1-ADP structure, to the surface-exposed position present in the apo-hIRE1 structure. It has been previously hypothesized that the non-productive position of the αC-helix in human and mouse IRE1 structures region may prohibit back-to-back dimer formation [24]. Back-to-back dimer formation requires changes in the region spanning the C-terminal end of αC-helix and the N-terminal end of strand β4, which might be coupled to conformational changes in Tyr628 (Figure 4B).

We compared the activities of WT and Tyr628Phe mutant IRE1 in two further biochemical assays that measured peptide substrate phosphorylation and RNase activity (Figure S1B, C). In contrast to the results of the autophosphorylation assay, the two variants of IRE1 were indistinguishable in the peptide activity assay (Figure S1B). This may have been due to technical reasons, such as the different experimental conditions that were required for this assays or may reflect a genuine disconnection between the kinetics of IRE1 autophosphorylation and peptide substrate phosphorylation. In the RNase assay, double the concentration of WT IRE1 was required to obtain the same signal as for Tyr628Phe, and the mutant thus appears to have a higher specific activity (Figure S1C). This is consistent with more rapid autophosphorylation of the Tyr628Phe, although differences between the experimental conditions of the RNase and autophosphorylation assays preclude a direct comparison of the kinetics of these two activities.

### Comparison of human and yeast IRE1 back-to-back dimers

**Figure 5:**
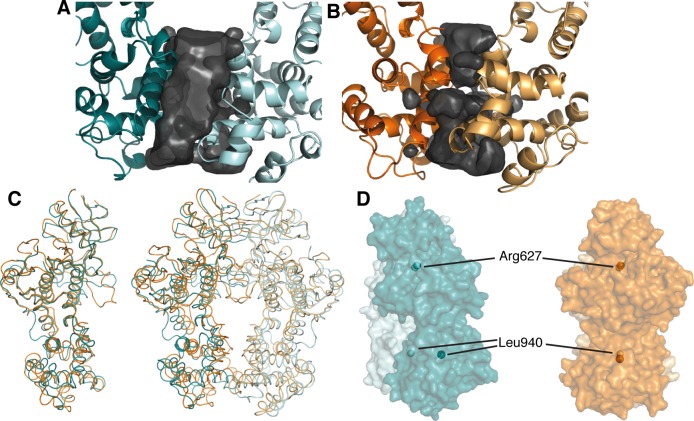
The apo-hIRE1 dimer is twisted compared to the phos-yIRE1 dimer, and the RNase domains are further apart **A.** & **B.** Voids at the IRE1 RNase dimer interface are shown as a black surface. Structures are shown in cartoon representation and individual chains are colored different intensities; **A.** apo-hIRE1; **B.** phos-yIRE1. Voids generated by HOLLOW v1.2 [42]. **C.** Cα traces showing superposition of apo-hIRE1 (blue) and phos-yIRE1 (orange) structures. Left, Aligned over a single monomer there is good correspondence of secondary structure elements (overall 1.17 Å Cα RMSD). Right, Aligned over the dimer the correspondence of secondary structure elements is less good, especially in the RNase domain (overall 3.79 Å Cα RMSD). **D.** Side view of the apo-hIRE1 and phos-yIRE1 (PDB ID 3FBV) dimers [18], colored as in A & B. Specific conserved residues are indicated by spheres; Arg617 in the N-lobe interface and Leu940 within the RNase domain (hIRE1 numbering). Additional views are shown in Figure S2.

Perhaps the most striking difference between the human and yeast IRE1 back-to-back dimer structures is the relatively weak engagement of the RNase domains within the unphosphorylated human dimer. The RNase domains of yIRE1 exhibit extensive contacts at the dimer interface, burying 703 Å^2^ of surface area [18, 20, 21]. However, in the apo-hIRE1 structure there is a large void between the RNase domains and only 227 Å^2^ is buried (Figure 5A, 5B). The difference at the RNase domain interface is not caused by large-scale intramolecular motion as the monomers in the apo-hIRE1 structure are almost identical to those of yIRE1 (Cα RMSD = 1.17 Å). However, the overall fit for the dimers is poorer (Cα RMSD = 3.79 Å) so the differences observed at the RNase interface must be caused by distinct relative orientations between protomers (Figure 5C, 5D). In the structures of yIRE1, the monomers are approximately parallel. However, in the apo-IRE1 structure there is a rotation of approximately 13° between protomers pivoting about a fulcrum at the interface in the N-lobe (Figure 5D, S2). The rotation keeps the N-lobes in close contact but leads to a ~ 10 Å displacement of residues within the RNase domain core.

**Figure 6:**
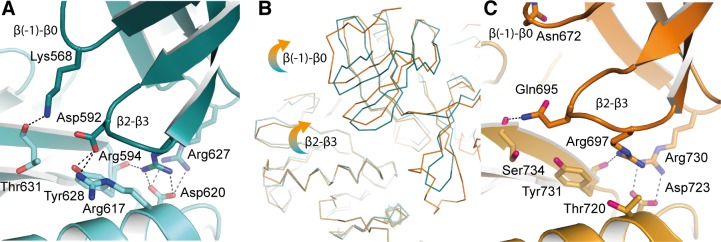
Differences in the back-to-back dimer contacts between hIRE1 and yIRE1 **A.** Apo-hIRE1 N-lobe dimer interface shown in cartoon representation with selected side chain and main chain atoms shown. Salt bridges and hydrogen bonds are shown as dashed black lines. Atoms are colored by element/chain: carbon, teal/pale blue; oxygen, red; nitrogen, blue. **B.** Ribbon representation of apo-hIRE1 and phos-yIRE1. The dimers are aligned over one chain – bottom left of panel. Perturbations in secondary structure of the opposing monomer can be seen. **C.** Phos-yIRE1 N-lobe dimer interface shown in the same representation as A (PDB ID 3FBV) [18]. Carbons in respective chains are colored light and dark orange.

The contacts between monomers at the apo-hIRE1 dimer interface are symmetrical and mainly within the N-lobe. An extensive network of salt bridges and hydrogen bonds is formed between an acidic region at the C-terminal end of αC and β4 on one protomer and a basic protrusion formed by the β(−1)-β0 and β2-β3 loops on the opposing monomer (Figure 6A, 6B, S3A, S3B). In yIRE1, with the activation loop phosphorylated, there are fewer interactions in the N-lobe and those which are made are between different residues even though the residues are identical or similar (Figure 6C, S4). For example, in apo-hIRE1 Arg594 forms an ion pair with Asp620 and a hydrogen bond to Tyr628 carbonyl while the equivalent residue in yIRE1 (Arg697) is displaced and can no longer form the salt-bridge (Figure 6A, 6C). The secondary structure elements are displaced and are coupled to a rotation at the dimer interface (Figure 5D, 6B) that disengages the RNase domains of the two monomers, relative to the positions observed in the phos-yIRE1 structure.

Another difference between phos-yIRE1 and apo-hIRE1 back-to-back dimers is the presence of an additional α-helix (termed αE′ in yIRE1 [20]) between β7 and β8 in the kinase C-lobe in the phos-yIRE1 structure (Figure S3C, D). This contributes substantially to the interface of the yIRE1 back-to-back dimer, but only contributes 2 hydrogen bonds over the equivalent loop region in human IRE1. Twisting of the hIRE1 back-to-back dimer interface to more closely resemble the yIRE1 interface would probably increase the number of contacts, but there is no scope for the contacts to contribute as much as in the yIRE1 structure.

### The structure of hIRE1 bound to a kinase inhibitor/RNase activator

**Figure 7:**
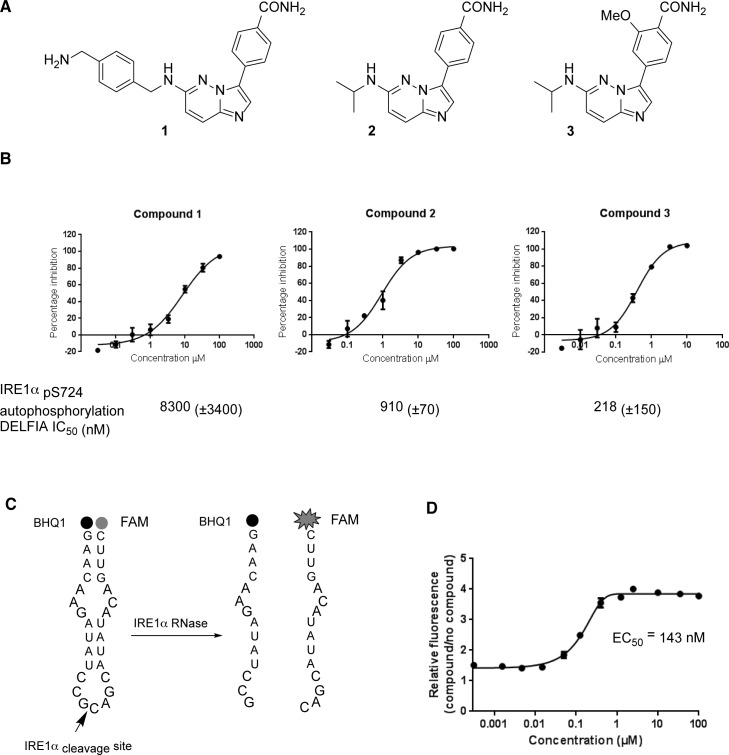
Chemical synthesis and biological activity of a human IRE1 kinase inhibitor that stimulates RNase activity **A.** Chemical structures of compounds 1, 2 and 3. **B.** Compounds 1, 2 and 3 inhibit the *in vitro* autophosphorylation of hIRE1α; representative curves shown, IC50 (±SD), *n* > 3 determinations. **C.** A 29-mer stem-loop RNA is cleaved specifically by hIRE1α in a FRET assay format to measure inhibition or activation of hIRE1α RNase function [29]. **D.** Kinase inhibitor 3 enhances hIRE1α RNase cleavage of the stem-loop RNA substrate *in vitro*.

We developed a series of hIRE1 kinase inhibitors based on a 4-(imidazo [1,2-*b*]pyridazin-3-yl)benzamide scaffold using microwave-assisted reactions (Figure 7A, 7B, S5) [29, 30]. Among these compounds was the sub-micromolar hIRE1 kinase inhibitor 3. Compound 3 was assessed for the ability to inhibit or enhance the endoribonuclease function of hIRE1 (Figure 7C, 7D). This assay clearly showed that compound 3 enhanced the endoribonuclease activity of unphosphorylated hIRE1 *in vitro* with potency (EC_50_ 143 nM), similar to the inhibition of the kinase autophosphorylation activity (IC_50_ 218 nM).

**Figure 8:**
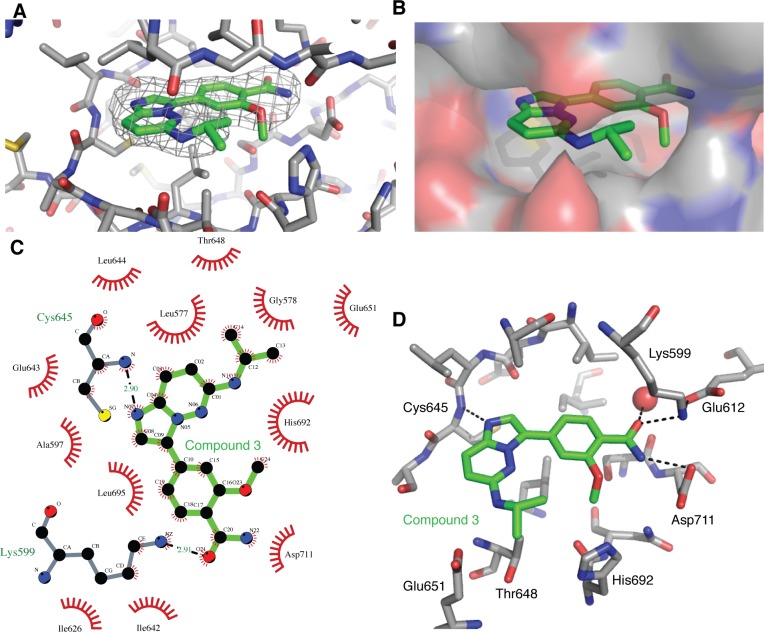
Binding of compound 3 to the IRE1 kinase active site **A.** Compound 3 (green carbon atoms) is located in the hIRE1 ATP binding pocket (grey carbon atoms). Wire mesh shows simulated annealing omit electron density map after removal of ligand from final model. **B.** Same view as A, but with hiRE1 shown as a translucent surface. **C.** Summary of the interactions generated using Ligplot+ [43]. Red flashes show Van der Waal contacts, H-bonds are marked with black dashed lines and distances in Å. **D.** Key protein-ligand interactions in the crystal structure. Black dashed lines are potential H-bonds. Red sphere is an ordered water molecule that mediates interactions between the ligand amide group and the protein DFG motif.

The compound (3) was soaked into apo-hIRE1 crystals and the structure of the complex was determined to 2.9Å. The difference electron density due to the compound 3 was clear and the ligand was modeled in a single position and in an unambiguous orientation (Figure 8A). The active site is closed up around the ligand and Van der Waal contacts are made with almost all of its atoms (Figure 8B, 8C). The ligand has a classical binding mode, forming a single H-bond with the kinase hinge region between the imidazole *N*-1 atom and the main chain NH of Cys645 (Figure 8C, 8D). The isopropylamino group is sandwiched between Gly578 (P-loop), Thr648/Glu651 (αD helix) and His692. The methoxybenzamide moiety extends towards the hIRE1 DFG motif, and the amide group forms a network of interactions with the side chain of Asp711 (DFG), Lys599 and Glu612 (Lys-Glu salt bridge), and a buried water molecule.

**Figure 9:**
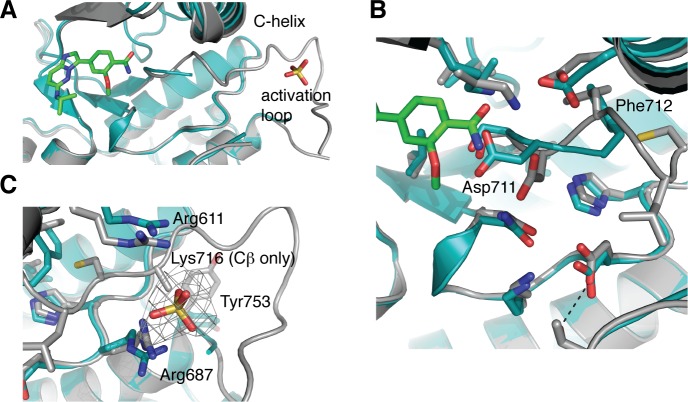
hIRE1 in complex with compound 3 has a more ordered activation loop **A.** Superposition of apo-hIRE (teal) and ligand-bound hIRE1 (grey). Compound 3 and a sulfate ion are shown as sticks. The activation loop of apo-hIRE1 is mostly disordered. **B.** Magnified view in the vicinity of the DFG motif. In the presence of ligands, Asp711 and Phe712 adopt the positions found in active kinase structures. **C.** Magnified view in the vicinity of the sulfate ion. The wire mesh shows simulated annealing omit electron density map after removal of sulfate from final model. All structure figures were generated in PyMOL [44].

Comparison of the apo and ligand-bound hIRE1 structures shows that most of the active site residues can be superimposed (Figure 9A). However, there are clear differences between the positions of the DFG motif and the Lys-Glu salt bridge. The position of the Asp711 side chain in the apo-hIRE1 structure is incompatible with inhibitor binding and this residue moves to accommodate the ligand (Figure 9B). Furthermore the orientation of Phe712 side chain has altered such that the R-spine is fully intact. The activation loop is well defined from 711-726 and 732 onwards in the ligand-bound structure, whereas residues 713-731 are unresolved in the apo structure. This may partly be explained by the presence of a sulfate ion in the vicinity of Arg687 (HRD motif) and Arg611 (αC-helix) that may help to organize the activation loop by mimicking the phosphorylation of the activation loop (Figure 9C).

## DISCUSSION

**Figure 10:**
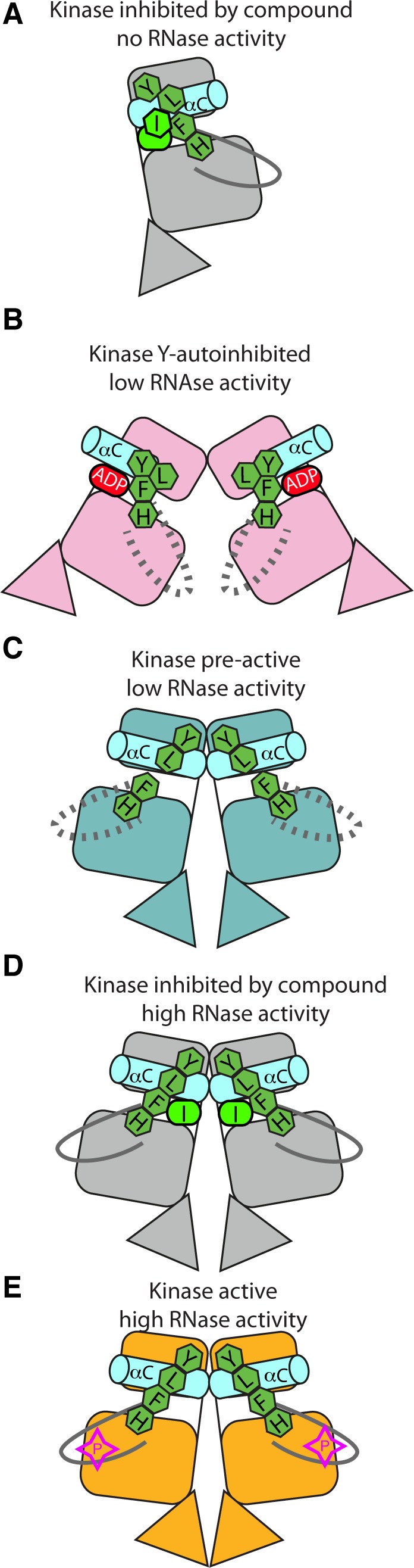
Graphical summary of IRE1 structures **A.** hIRE1 crystallizes as a monomer in the complex with a sulfonamide inhibitor (PDB code 4U6R). The R-spine is disrupted by a chlorophenyl group that pushes the αC-helix out of position. **B.** ADP-hIRE1 crystallizes as a face-to-face dimer (PDB code 3P23). The monomers are in a conformation in which autophosphorylation is inhibited. The down position of Tyr628 breaks the R-spine, and αC-helix is rotated away from the N-lobe to generate a surface that cannot form the back-to-back dimer. **C.** apo-hIRE1 crystallizes as a back-to-back dimer. Tyr628 is in the up position, the αC-helix is in an active position, but the R-spine is not fully formed. **D.** The structure of hIRE in complex with a kinase inhibitor that stimulates RNase activity (I) has a more ordered activation loop and a fully-formed R-spine. **E.** phospho-yIRE1 crystallizes as a back-to-back dimer in which the activation loop is fully ordered, and the R-spine is intact (PDB code 3FBV). Full RNase activity depends on multimerisation of yIRE1 protomers (not shown).

IRE1 has been captured in crystal structures that might represent steps along the activation pathway (Figure 10). States of no or low RNase activity exhibit crystal structures in which the kinase domains have inactive conformations characterized by one or more of the following features: broken R-spine, distorted αC-helix and disordered activation loops (Figure 10A, 10B, 10C). States of high RNase activity exhibit crystal structures in which the kinase domain has an active conformation (Figure 10D, 10E). It is debatable whether the present back-to-back dimer structure represents the RNase active state which, using the yeast protein as a model, most likely requires phosphorylation and multimerisation [18]. This final state of the human protein has thus far eluded attempts at crystallization, but it may succumb to a sustained attack using a combination of carefully controlled protein phosphorylation protocols and co-crystallization using ligands that enhance RNase activity.

### Conservation of IRE1 back-to-back dimerization in yeast and humans

The structure of apo human IRE1 confirms that the human protein can adopt a back-to-back conformation previously only seen in the yeast protein, consistent with previous experiments showing that point mutations in the back-to-back interface are required for RNase activity in the highly similar murine protein [24]. From this we conclude that hIRE1 can form an RNase active site similar to yIRE1, and that there is a conserved activation mechanism that couples IRE1 kinase and RNase activities. Our structures of hIRE1 revealed that the protomers were twisted with respect to each other whereas the protomers of phos-yIRE1 are parallel. In support of the notion that this observation could represent an important intermediate in the activation process of hIRE1, each of the two crystal forms has two hIRE1 molecules in the asymmetric unit that have different packing arrangements. Autophosphorylation increases the RNase activity of IRE1 cytoplasmic region, even when constitutively dimerised [31]. The twist in apo-hIRE1 disengages the RNase domains, which might reflect lower RNase activity. However, the significance of the relative orientation of back-to-back interfaces between hIRE1 and yIRE1 is presently unclear. Indeed, there are several differences between the structures that might explain the twisted interface in the hIRE1 structure: the absence of phosphorylation on the activation loop, differences in crystal packing, lack of ligand occupancy in the kinase active site, or differences between the primary sequences. Recent structures of the IRE1 homologue RNase L revealed a greater angle between protomers than we have seen for apo-hIRE1 while maintaining an RNase active conformation of RNase L [32, 33]. RNase L has a C-terminal pseudokinase/ribonuclease domain homologous to IRE1 however it has a N-terminal ankyrin repeat domain which differs from IRE1. RNase L dimerization is driven by binding a small molecule nucleotide metabolite in the cavity between the ankyrin repeat domain and the N-lobe. Although there is a large twist between pseudokinase protomers, the RNase domains in RNase L are well engaged and form a well-ordered RNase active site. To accommodate the twist between protomers, the N-lobes are shifted compared to the IRE1 structures but still make intermolecular contacts. The RNase L dimer is further stabilized by an extensive amount of buried surface area at the dimer interface in the ankyrin repeat domain.

### Kinase active site ligands that enhance or inhibit IRE1 RNase

The IRE1-XBP1 pathway has emerged as a promising target in multiple myeloma and triple negative breast cancers amongst other diseases [11, 16]. Structure-based drug-design programs can exploit unliganded hIRE1 crystal forms to advance therapeutic progress, although a key challenge will be to develop compounds that potently inhibit kinase activity without stimulating XBP1 splicing. In this present study, we have shown that the IRE1 kinase inhibitor imidazopyridazine compound 3 binds to the ATP binding site, stabilizes an active conformation of the kinase, including an intact R-spine, and is an enhancer of RNase activity. This data supports the hypothesis that IRE1 kinase inhibitors that stimulate the RNase activity of human IRE1, such as compound 3 and APY29, act through stabilization of an active conformation of the kinase active site [12, 24]. In contrast, a kinase inhibitor that also inhibits RNase activity displaces key active site residues, disrupts the R-spine and distorts the αC-helix (Figure 10A) [26]. Type II inhibitors, widely studied in other kinases, displace the DFG motif, thus disrupting the R-spine, and although structural data have not been reported, an exemplar of this class of IRE1 kinase inhibitor has demonstrated RNase inhibition [12]. Furthermore, ADP has been described as an IRE1 RNase inhibitor and the autoinhibitory Tyr-down conformation in the ADP-bound hIRE1 structure also disrupts the R-spine and distorts the αC-helix (Figure 10B) [17, 22, 24]. It is likely that structural biology will play a key role in aiding the design of highly selective and potent IRE1 inhibitors with the desired pharmacological profile.

### Kinase autoinhibition through a Tyr residue on strand β4

The autoinhibitory mechanism present in IRE1 is mediated by a tyrosine residue at the N-terminus of β4, which is present in approximately 10% of the human kinome. An equivalent mechanism was first identified in Nek7, a kinase that can be activated by an upstream kinase or by autophosphorylation through an intermolecular mechanism [27, 34]. In both cases, the mechanism serves to prevent untimely self-activation. Further work will be required to identify which other kinases are regulated through a similar autoinhibitory conformation, and whether formation of a back-to-back dimer provides a general mechanism for release of the tyrosine, leading to kinase activation.

## MATERIALS AND METHODS

### Cloning and protein production

Large scale production of wt hIRE1 was performed as in Ref. [29]. Briefly, hIRE1 residues Gly547-Leu977 was cloned into a modified version of pFastBac which contains an N-terminal 6-His tag followed by a rhinovirus 3C protease site. *Spodoptera frugiperda (Sf9)* cells were grown to a cell density of 2 × 10^6^ cell/mL and infected with ~50 μL of virus per 10^7^ cells. Cultures were harvested after 3 days.

To create hIRE1 mutants, the same IRE1 fragment was subcloned into a modified version of pMAX (Lonza) providing an N-terminal tandem Strep2 tag, FLAG tag and rhinovirus 3C protease site. Mutants were created by the Quikchange method (Stratagene). Hek293 suspension cells were transfected at 10^6^ cell/mL with 0.5 μg DNA per mL and 2 μg/mL polyethyleneamine. Cells were harvested 48hrs after transfection.

### Protein purification

His-hIRE1 cell pellet was resuspended in 4 volumes of 200 mM NaCl, 50 mM HEPES (pH 7.5), 10% glycerol, 1 mM CaCl_2_, 1 mM MgCl_2_, 80 U/mL DNase I and 1 EDTA-free protease inhibitor tablet (Roche). Cells were lysed by sonication and clarified by centrifugation at 45,000*g* for 40 min at 4°C followed by sequential filtration through 1.2 μm and 0.45 μm filters before application to 10 mL TALON resin (Clontech). IRE1 was purified using gravity flow. The column was washed 3 times with 5 column volumes of 200 mM NaCl, 50 mM HEPES (pH 7.5) and 10% glycerol with 2.5 mM or 5 mM imidazole. Proteins were eluted in 20 mL 200 mM NaCl, 50 mM HEPES (pH 7.5), 10% glycerol and 250 mM imidazole. 0.04 U/pmol of λ-phosphatase (NEB) and 300 μg of GST tagged-rhinovirus 3C protease were added to purified proteins which were then dialyzed against 200 mM NaCl and 50 mM HEPES (pH 7.5) overnight at 4°C. The ionic strength of the sample was reduced by dilution with 50 mM HEPES (pH 7.5) to obtain a final concentration of NaCl ≈ 40 mM. The sample was filtered through a 0.45 μm filter before application to a 5 mL Q-HP Trap (GE Healthcare) and was purified on a gradient to 1 M NaCl and 50 mM HEPES (pH 7.5). As a final purification step, proteins were purified by size exclusion chromatography on a 16/60 Superdex 200 column (GE Healthcare) in 200 mM NaCl, 50 mM HEPES (pH 7.5), 2 mM EDTA and 5 mM DTT.

Purification of IRE1 mutants followed a similar protocol. Cells were lysed by sonication in 200 mM NaCl, 50 mM HEPES (pH 7.5), 1 mM MgCl_2_, 1 mM CaCl_2_ and 1 EDTA-free protease inhibitor tablet (Roche). Samples were clarified at 45,000*g* for 40 min at 4°C. Proteins were passed through 0.45 μm filter applied to a 1 mL StrepTrap (GE Healthcare), washed with 10 mL 200 mM NaCl and 50 mM HEPES (pH 7.5) and eluted in 200 mM NaCl, 50 mM HEPES (pH 7.5) and 2.5 mM desthiobiotin. Tag removal and protein dephosphorylation was performed as above. 3C protease was removed by flowing the mixture through a 1mL GSTrap (GE Healthcare) before sample concentration and purification by size exclusion chromatography using a 16/60 Superdex 200 column in to 200 mM NaCl, 50 mM HEPES (pH 7.5), 2 mM EDTA and 5 mM DTT. Proteins were concentrated in centrifugal concentrators (Vivaspin) prior to freezing in liquid N_2_ and storage at −80°C.

### Protein crystallization and crystallography

Apo-hIRE1 was crystallized by hanging-drop vapor diffusion by mixing IRE1 (at 2 mg/mL) with reservoir solution containing 18-20% PEG3350, 0.2 M sodium malonate and 0.1 Bis-Trispropane (pH 6.5) in a 1:1 ratio. Crystallization experiments were conducted at 18°C. Crystals also grew if sodium malonate was replaced with sodium sulfate, or K/Na tartrate. Single crystals grew more reproducibly when crystallization drops were micro-seeded using a cat whisker, otherwise crystals grew in joined clusters. Crystals were briefly (≈30 sec) soaked in 22% PEG3350, 0.2 M Na Malonate, 0.1 M Bis-Trispropane (pH 7.5) and 25% ethylene glycol before plunging into liquid N_2_.

To prepare crystals for compound soaking, hIRE1-apo crystals were grown in 20% PEG 3350, 0.2M NaSO_4_, 0.1 M Bis-Trispropane (pH 6.5). A single crystal was transferred to solution supplemented with 0.1 mM compound 3 for 20 hours, briefly soaked in the same buffer supplemented with 20% ethylene glycol, and then flash cooled in liquid N_2_.

X-ray diffraction data were collected at 100 K from single cryo-cooled hIRE1 crystals at Beamline I04-1 at DIAMOND Lightsource. Diffraction data were integrated using the XDS software package [35], and scaled and merged using AIMLESS [36] within CCP4 [37]. The structure of apo-hIRE1 was solved by molecular replacement using Phaser 2.1 [38] with a single protomer from the ADP-hIRE1 crystal structure (PDB ID 3P23) [22]. The ligand-bound structure was solved by molecular replacement, using apo-hIRE1 as the model. Manual model building was performed in COOT [39] and refinement was performed in Buster 2.1 with NCS restraints [40, 41].

### Autophosphorylation assay

IRE1 autophosphorylation activity and compound inhibition (IC_50_) was measured using a Dissociation-Enhanced Lanthanide Fluorescent Immunoassay (DELFIA) assay as in Ref. [29]. Briefly, dephosphorylated IRE1 was incubated for 25 min with 100 μM ATP in 15 μL assay buffer (40 mM Tris (pH 7.5), 20 mM MgCl_2_ and 1 mM DTT). The assay was stopped by the addition of 40 mM EDTA. Samples were transferred to a 384-well high-binding plate and incubated overnight at 4°C. Plates were washed three times in 0.1% Tween-20 followed by blocking in 5% non-fat skimmed milk in PBS for 30 mins at 37°C. Plates were washed again before application of α-pS724 phospho-specific IRE1 primary antibody [29] (160 pg/mL) in PBS to each well and incubated for 1.5 hour are 37°C. The plates were washed before addition of a Europium labeled α-rabbit secondary antibody (Perkin Elmer Life Sciences). Another wash step was followed by the addition of DELFIA enhancement solution (Perkin Elmer Life Sciences). Assay plates were read on an EnVision 2103 microplate reader (Perkin Elmer Life Sciences).

### Substrate phosphorylation assay

Peptide phosphorylation was measured using the STK Substrate 2-biotin (Biotinyl-Ahx-RRRLSFAEPG-CONH2) substrate from the HTRF KinEASE kit (Cisbio, MA, USA). Assays were carried out in low volume, black 384-well plates (3676 from Corning Life Sciences, MA), with a 10 μl assay volume containing 30 μM ATP (Km = 26 μM), 600 nM STK Substrate 2-biotin and 50 nM of dephosphosphorylated Ire1. Following incubation at 37°C over the time course, the reaction was stopped with buffered EDTA, which contained the detection reagents, streptavidin-XL665 and the STK-antibody labelled with Eu^3+^-cryptate. The resulting TR-FRET signal, calculated as the fluorescence ratio at 665/620 nm, was read on an Envision and was proportional to the level of phosphorylation of the peptide.

### Endoribonuclease assay

IRE1 endoribonuclease activity was measured using a FRET derepression assay monitoring cleavage of a 29-nucleotide stem-loop RNA containing the XBP1 cleavage site sequence and labelled with a fluorescence emitter (FAM) and a fluorescence quencher (BHQ) at the 5′ and 3′ ends, respectively [29] (Figure 7C). Upon cleavage of the stem-loop, dissociation of the product strands leads to derepression of the FRET between emitter and quencher, and signal is recovered. Briefly, varying volumes of compound in DMSO or DMSO alone were added to a low volume 384 well plate (3676, Corning, USA) to give final concentrations ranging from 100 μM to 0.313 nM using an Echo acoustic liquid dispenser (Labcyte, CA., USA). Non-phosphorylated IRE1 G547-L977 was added to a final concentration of 200 nM. After incubating for 30 minutes at 30^o^ C, hairpin RNA XBP-1 substrate mimic labelled with fluorescein and Black Hole quencher (5′ FAM-GAACAAGAUAUCCGCA-GCAUAUACAGUUC-3′ BHQ, Eurofins MWG Operon, Germany) was added to 100 nM final concentration. After incubating for a further 15 minutes at 30°C, the fluorescein fluorescence was measured on an EnVision plate reader (Perkin-Elmer, MA., USA). Fluorescence in the presence of compound was expressed relative to that of DMSO alone (no compound).

### Chemistry methods

Anhydrous solvents and reagents were used as obtained from commercial suppliers. Flash column chromatography was carried out on Merck silica gel 60 (0.040-0.063 mm). Analytical thin layer chromatography (TLC) was performed on pre-coated aluminium sheets of silica (60 F254, Merck) and visualised by short-wave UV light and permanganate dip. ^1^H-Nuclear magnetic resonance spectra were recorded at 500 MHz on Bruker AMX500 spectrometers using an internal deuterium lock. Chemical shifts were measured in parts per million (ppm) relative to tetramethylsilane (δ = 0) using the following internal references for residual protons in the solvent: CD_3_OD (δH 3.32) and (CD_3_)_2_SO (δH 2.50). Coupling constants (J) are quoted to the nearest 0.5 Hz. ^13^C-nuclear magnetic resonance spectra were recorded at 126 MHz on Bruker AMX500 spectrometers using an internal deuterium lock. All chemical shift values were reported in ppm relative to tetramethylsilane (δ = 0). The following internal references were used: CD_3_OD (δC 49.0) and (CD_3_)_2_SO (δC 39.5). LC/MS and HRMS analysis was performed on an Agilent 1200 series HPLC and diode array detector coupled to a 6210 time of flight mass spectrometer with dual multimode APCI/ESI source. Analytical separation was carried out at 30°C on a Merck Purospher STAR column (RP-18e, 30 × 4 mm) using a flow rate of 1.5 mL/min in a 4 minute gradient elution with detection at 254 nm. The mobile phase was a mixture of methanol (solvent A) and water containing formic acid at 0.1% (solvent B). Gradient elution was as follows: 1:9 (A/B) to 9:1 (A/B) over 2.5 min, 9:1 (A/B) for 1 min, and then reversion back to 1:9 (A/B) over 0.3 min, finally 1:9 (A/B) for 0.2 min. The references used for HRMS analysis were: caffeine [M+H]+ 195.087652; hexakis (2,2-difluroethoxy)phosphazene [M+H]^+^ 622.02896 and hexakis(1H,1H,3H-tetrafluoropentoxy)phosphazene [M+H]^+^ 922.009798.

3-Bromo-6-chloroimidazo [1,2-b]pyridazine (0.250 g, 1.07 mmol) and isopropylamine (0.366 mL, 4.30 mmol, 4 equiv.) in N-methylpyrrolidin-2-one (2.2 mL) were heated in a microwave reactor (Biotage) at 180°C for 3 h. The reaction was cooled, diluted with water (30 mL) and extracted with ethyl acetate (30 mL). The organic phase was washed with brine (3 × 20 mL), dried over magnesium sulfate, concentrated and the resulting crude product purified by ion exchange chromatography on acidic resin (5 g; Isolute SCX-2), eluting sequentially with methanol and then 1 M ammonia in methanol, to give 3-bromo-N-isopropylimidazo [1,2-b]pyridazin-6-amine (0.243 g, 0.95 mmol, 89%). ^1^H NMR (500 MHz, CD_3_OD) δH 7.52 (1H, d, J = 9.5 Hz), 7.39 (1H, s), 6.66 (1H, d, J = 9.5 Hz), 4.09 (1H, septet, J = 6.5 Hz), 1.29 (6H, d, J = 6.5 Hz); ^13^C (126 MHz, CD_3_OD) δC 153.8, 136.7, 129.4, 124.0, 113.7, 99.9, 42.5, 21.0; LC-MS (ESI+) m/z 253.03 [M+H^+^], R_t_ = 2.51 min; HRMS [M+H^+^] calcd. for C_9_H_12_Br^79^N_4_ 255.0240; found 255.0239. 3-Bromo-N-isopropylimidazo [1,2-b]pyridazin-6-amine (0.077 g, 0.30 mmol), 4-carbamoylphenylboronic acid (0.074 g, 0.45 mmol, 1.5 equiv.) and tetrakis(triphenylphosphine) palladium (0) (0.017 g, 0.015 mmol, 0.05 equiv.) in 2 M aqueous sodium carbonate (0.45 mL, 0.90 mmol, 3 equiv.) and dimethoxyethane (1.50 mL) were heated in a microwave reactor (Biotage) at 135°C for 1 h. The reaction mixture was cooled and passed through an acidic ion exchange column (5 g; Isolute SCX2) washing sequentially with methanol, and 1 M ammonia in methanol. Purification of the crude product by silica gel chromatography, eluting with 5-20% methanol in dichloromethane, gave 4-(6-(isopropylamino)imidazo [1,2-b]pyridazin-3-yl)benzamide (2; 0.068 g, 0.23 mmol, 77%). ^1^H NMR (500 MHz, (CD_3_)_2_SO) δH 8.31-8.28 (2H, m), 7.99 (2H, br s), 7.97-7.95 (2H, m), 7.75 (1H, d, J = 9.5 Hz), 7.34 (1H, br s), 6.98 (1H, d, J = 6.5 Hz), 6.70 (1H, d, J = 9.5 Hz), 3.96 (1H, apparent octet, J = 6.5 Hz), 1.26 (6H, d, J = 6.5 Hz); ^13^C (126 MHz, (CD_3_)_2_SO) δC 167.9, 153.3, 138.0, 132.6, 132.5, 131.2, 128.2, 126.4, 126.1, 125.3, 113.2, 43.0, 22.4; LC-MS (ESI+) m/z 296.17 [M+H^+^], R_t_ = 1.88 min; HRMS [M+Na^+^] calcd. for C_16_H_17_N_5_NaO 318.1325; found 318.1319.

Dimethyl formamide (3.26 mL) was added to methyl 4-bromo-2-methoxybenzoate (0.200 g, 0.816 mmol), potassium acetate (0.240 g, 2.448 mmol, 3 equiv.) and bis(pinacolato)diboron (0.311 g, 1.224 mmol, 1.5 equiv.). Nitrogen was bubbled through the mixture for 10 min, followed by addition of palladium chloride (diphenylphosphineferrocene) dichloromethane complex (0.033 g, 0.041 mmol, 0.05 equiv.). The reaction mixture was heated at 100°C in a microwave reactor (Biotage) for 90 min. The cooled mixture was partitioned between brine (140 mL) and ethyl acetate (100 mL). The organic layer was further washed with brine (50 mL), dried over magnesium sulfate and evaporated to dryness. The resulting crude methyl 2-methoxy-4-(4,4,5,5-tetramethyl-1,3,2-dioxaborolan-2-yl)benzoate was used in the next step without further purification. LC-MS (ESI+) m/z 293.21 [M+H^+^], R_t_ = 2.74 min. Dimethoxyethane (3.1 ml) and 2 M aqueous sodium carbonate (0.94 ml, 1.880 mmol) were added to 3-bromo-N-isopropylimidazo [1,2-b]pyridazin-6-amine (0.160 g, 0.627 mmol) and methyl 2-methoxy-4-(4,4,5,5-tetramethyl-1,3,2-dioxaborolan-2-yl)benzoate (0.238 g, 0.815 mmol). Nitrogen was bubbled through for 10 min followed by addition of tetrakis(triphenylphosphine)palladium (0) (0.036 g, 0.031 mmol). The reaction mixture was heated at 135°C in a microwave reactor (Biotage) for 1 h. The cooled reaction mixture was diluted with methanol and purified by ion exchange column chromatography on acidin resin (5 g; Isolute SCX2), eluting sequentially with methanol, then 2M ammonia in methanol. The combined basic fractions were further purified by silica column chromatography, eluting with 2% methanol in dichloromethane, to give methyl 4-(6-(isopropylamino)imidazo [1,2-b]pyridazin-3-yl)-2-methoxybenzoate (141 mg, 66%). ^1^H NMR (500 MHz, CD_3_OD) δH 8.19 (1H, d, J = 1.5 Hz), 7.92 (1H, s), 7.83 (1H, d, J = 8.0 Hz), 7.67 (1H, dd, J = 8.0, 1.5 Hz), 7.61 (1H, d, J = 9.5 Hz), 6.70 (1H, d, J = 9.5 Hz), 4.12 (1H, apparent octet, J = 6.5 Hz), 3.98 (3H, s), 3.89 (3H, s), 1.31 (6H, d, J = 6.5 Hz); ^13^C NMR (126 MHz, CD_3_OD) δC 166.7, 159.5, 153.3, 137.9, 135.0, 131.4, 129.8, 126.6, 124.4, 117.2, 117.1, 113.7, 109.0, 55.0, 51.0, 42.6, 21.1; LC-MS (ESI+) m/z 341.27 [M+H^+^], R_t_ = 2.19 min; HRMS [M+H^+^] calcd. for C_18_H_21_N_4_O_3_ 341.1608; found 341.1610. Methyl 4-(6-(isopropylamino)imidazo [1,2-b]pyridazin-3-yl)-2-methoxybenzoate (0.029 g, 0.087 mmol) in 7 M ammonia in methanol (3.7 mL) was heated at 74°C for 3 days in a sealed vial. The reaction mixture was evaporated to dryness. The crude product was purified by preparative thin layer chromatography (500 microns silica gel), eluting with 5% methanol in dichloromethane, to give 4-(6-(isopropylamino)imidazo [1,2-b]pyridazin-3-yl)-2-methoxybenzamide (3; 24 mg, 84%). ^1^H NMR (500 MHz, (CD_3_)_2_SO) δH 8.19 (1H, d, J = 1.5 Hz), 8.04 (1H, s), 7.89 (1H, d, J = 8.0 Hz), 7.76 (1H, d, J = 9.5 Hz), 7.76-7.74 (1H, m), 7.66 (1H, br s), 7.53 (1H, br s), 7.02 (1H, br d, J = 7.0 Hz), 6.71 (1H, d, J = 9.5 Hz), 4.02 (1H, apparent octet, J = 6.5 Hz), 4.00 (3H, s), 1.26 (6H, d, J = 6.5 Hz); ^13^C NMR (126 MHz, (CD_3_)_2_SO) δC 166.3, 157.9, 153.3, 138.2, 134.0, 131.6, 131.5, 126.2, 126.1, 120.7, 117.9, 113.4, 108.7, 56.2, 42.8, 22.5; LC-MS (ESI+) m/z 326.30 [M+H^+^], R_t_ = 1.68 min; HRMS [M+H^+^] calcd. for C­_17_H_20_N_5_O_2_ 326.1612; found 326.1613.

## SUPPLEMENTARY MATERIALS FIGURES



## References

[1] Walter P., Ron D. (2011). The unfolded protein response: from stress pathway to homeostatic regulation. Science.

[2] Ron D., Walter P. (2007). Signal integration in the endoplasmic reticulum unfolded protein response. Nat Rev Mol Cell Biol.

[3] Sidrauski C., Walter P. (1997). The transmembrane kinase Ire1p is a site-specific endonuclease that initiates mRNA splicing in the unfolded protein response. Cell.

[4] Cox J.S., Shamu C.E., Walter P. (1993). Transcriptional induction of genes encoding endoplasmic reticulum resident proteins requires a transmembrane protein kinase. Cell.

[5] Shamu C.E., Walter P. (1996). Oligomerization and phosphorylation of the Ire1p kinase during intracellular signaling from the endoplasmic reticulum to the nucleus. EMBO J.

[6] Yoshida H. (2001). XBP1 mRNA is induced by ATF6 and spliced by IRE1 in response to ER stress to produce a highly active transcription factor. Cell.

[7] Gonzalez T.N. (1999). Mechanism of non-spliceosomal mRNA splicing in the unfolded protein response pathway. EMBO J.

[8] Kaser A. (2008). XBP1 links ER stress to intestinal inflammation and confers genetic risk for human inflammatory bowel disease. Cell.

[9] Cao S.S., Kaufman R.J. (2013). Targeting endoplasmic reticulum stress in metabolic disease. Expert Opin Ther Targets.

[10] Carrasco D.R. (2007). The differentiation and stress response factor XBP-1 drives multiple myeloma pathogenesis. Cancer Cell.

[11] Chen X. (2014). XBP1 promotes triple-negative breast cancer by controlling the HIF1alpha pathway. Nature.

[12] Wang L. (2012). Divergent allosteric control of the IRE1alpha endoribonuclease using kinase inhibitors. Nat Chem Biol.

[13] Volkmann K. (2011). Potent and selective inhibitors of the inositol-requiring enzyme 1 endoribonuclease. J Biol Chem.

[14] Papandreou I. (2011). Identification of an Ire1alpha endonuclease specific inhibitor with cytotoxic activity against human multiple myeloma. Blood.

[15] Cross B.C. (2012). The molecular basis for selective inhibition of unconventional mRNA splicing by an IRE1-binding small molecule. Proc Natl Acad Sci U S A.

[16] Mimura N. (2012). Blockade of XBP1 splicing by inhibition of IRE1alpha is a promising therapeutic option in multiple myeloma. Blood.

[17] Prischi F. (2014). Phosphoregulation of Ire1 RNase splicing activity. Nat Commun.

[18] Korennykh A.V. (2009). The unfolded protein response signals through high-order assembly of Ire1. Nature.

[19] Papa F.R. (2003). Bypassing a kinase activity with an ATP-competitive drug. Science.

[20] Lee K.P. (2008). Structure of the dual enzyme Ire1 reveals the basis for catalysis and regulation in nonconventional RNA splicing. Cell.

[21] Wiseman R.L. (2010). Flavonol activation defines an unanticipated ligand-binding site in the kinase-RNase domain of IRE1. Mol Cell.

[22] Ali M.M. (2011). Structure of the Ire1 autophosphorylation complex and implications for the unfolded protein response. EMBO J.

[23] Kornev A.P. (2006). Surface comparison of active and inactive protein kinases identifies a conserved activation mechanism. Proc Natl Acad Sci U S A.

[24] Sanches M. (2014). Structure and mechanism of action of the hydroxy-aryl-aldehyde class of IRE1 endoribonuclease inhibitors. Nat Commun.

[25] Korennykh A., Walter P. (2012). Structural basis of the unfolded protein response. Annu Rev Cell Dev Biol.

[26] Harrington PE, B. K., Malwitz D, Tasker AS, Mohr C, Andrews KL, Dellamaggiore K, Kendall R, Beckmann H, Jaeckel P, Materna-Reichelt S, Allen JR, Lipford JR (2014). Unfolded Protein Response in Cancer: IRE1 Inhibition by Selective Kinase Ligands Does Not Impair Tumor Cell Viability. ACS Med. Chem. Lett.

[27] Richards M.W. (2009). An autoinhibitory tyrosine motif in the cell-cycle-regulated Nek7 kinase is released through binding of Nek9. Mol Cell.

[28] Bayliss R. (2012). On the molecular mechanisms of mitotic kinase activation. Open Biol.

[29] Newbatt Y. (2013). Identification of autophosphorylation inhibitors of the inositol-requiring enzyme 1 alpha (IRE1alpha) by high-throughput screening using a DELFIA assay. J Biomol Screen.

[30] Stanovnik B, T. M., Drnovšek I (1981). 3-Bromoimidazo [1,2-b]pyridazine-bromine and 3-Bromo-6-chloroimidazo [1,2-b]pyridazine-bromine complexes; new brominating agents for organic compounds. Synthesis.

[31] Itzhak D. (2014). Multiple autophosphorylations significantly enhance the endoribonuclease activity of human inositol requiring enzyme 1alpha. BMC Biochem.

[32] Huang H. (2014). Dimeric structure of pseudokinase RNase L bound to 2-5A reveals a basis for interferon-induced antiviral activity. Mol Cell.

[33] Han Y. (2014). Structure of human RNase L reveals the basis for regulated RNA decay in the IFN response. Science.

[34] Dodson C.A. (2013). A kinetic test characterizes kinase intramolecular and intermolecular autophosphorylation mechanisms. Sci Signal.

[35] Kabsch W. (2010). Xds. Acta Crystallogr D Biol Crystallogr.

[36] Evans P.R., Murshudov G.N. (2013). How good are my data and what is the resolution?. Acta Crystallogr D Biol Crystallogr.

[37] Winn M.D. (2011). Overview of the CCP4 suite and current developments. Acta Crystallogr D Biol Crystallogr.

[38] McCoy A.J. (2007). Phaser crystallographic software. J Appl Crystallogr.

[39] Emsley P. (2010). Features and development of Coot. Acta Crystallogr D Biol Crystallogr.

[40] Blanc E. (2004). Refinement of severely incomplete structures with maximum likelihood in BUSTER-TNT. Acta Crystallogr D Biol Crystallogr.

[41] Smart O.S. (2012). Exploiting structure similarity in refinement: automated NCS and target-structure restraints in BUSTER. Acta Crystallogr D Biol Crystallogr.

[42] Ho B.K., Gruswitz F. (2008). HOLLOW: generating accurate representations of channel and interior surfaces in molecular structures. BMC Struct Biol.

[43] Laskowski R.A., Swindells M.B. (2011). LigPlot+: multiple ligand-protein interaction diagrams for drug discovery. J Chem Inf Model.

[44] Schrodinger, LLC (2010). The PyMOL Molecular Graphics System Version 1.3r1.

