# Supplementary material for: Molecular mechanisms of human IRE1 activation through dimerization and ligand binding
Source: Oncotarget. 2015 Apr 18;6(15):13019–35. doi: 10.18632/oncotarget.3864 (PMC4536996; doi:10.18632/oncotarget.3864)
Supplement: Supplementary file 1 [file oncotarget-06-13019-s001.pdf]

Molecular mechanisms of human IRE1 activation through dimerization and ligand binding

Supplementary materials

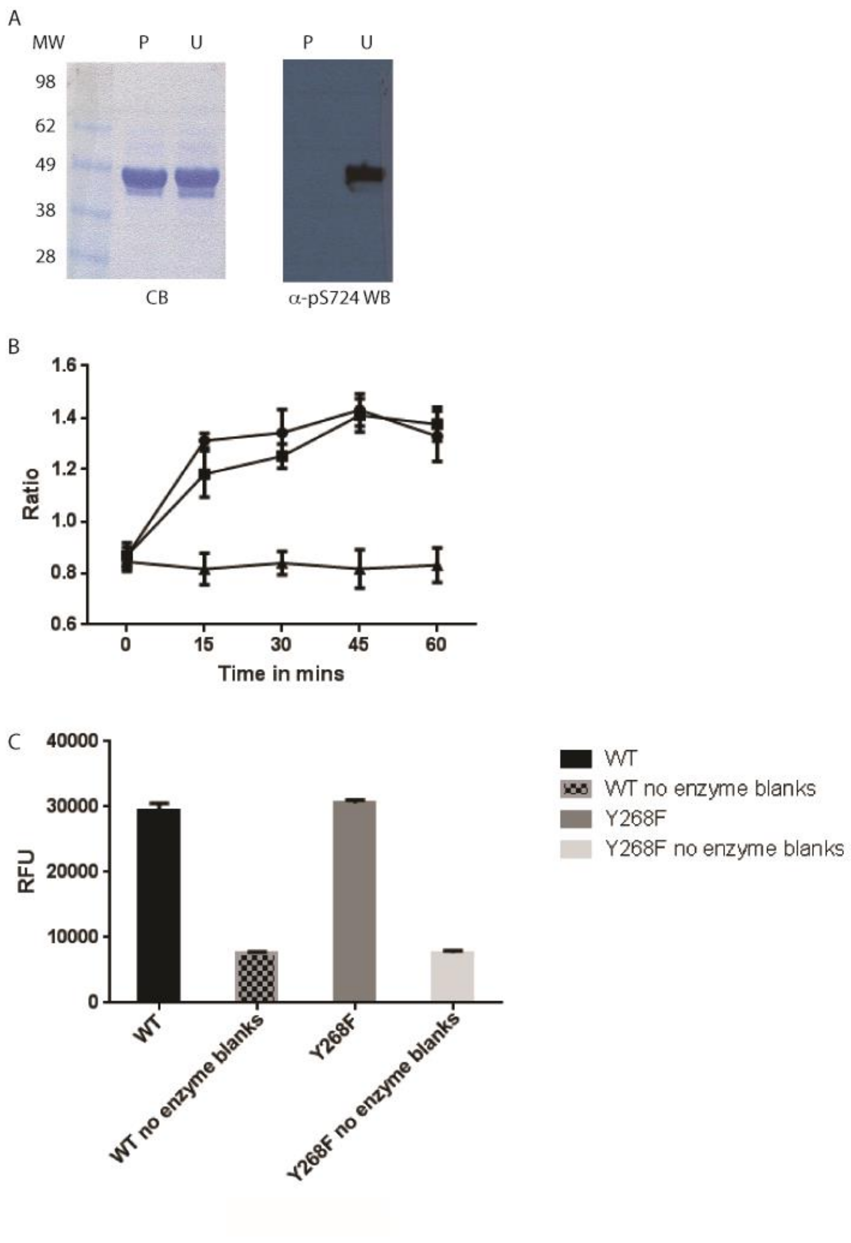

Figure S1: Biochemical characterization of hIRE1 547-977

(A) Western Blot 'WB' analysis of hIRE using phospho-specific S724 antibody to demonstrate complete dephosphorylation of protein preparatively treated with phosphatase (P), compared to untreated sample (U). Coomassie-stained gel is labeled 'CB'. (B) Time course of IRE1 WT (●) and Y628F mutant (■) at 50nM phosphorylating the generic substrate S2, STK Substrate 2-biotin (Biotinyl-Ahx-RRRLSFAEPG-CONH2) at 37°C in the presence of 30 µM ATP. No enzyme controls (▲). Results are mean ± SD (n=4). (C) RNase activity in the FRET depression assay. The dephosphorylated wild type (2000 nM) and Y268F mutant

(1000 nM), pre-incubated with 0.5 mM ATP for 45 min. Error bars represent standard deviation of 4 replicates.

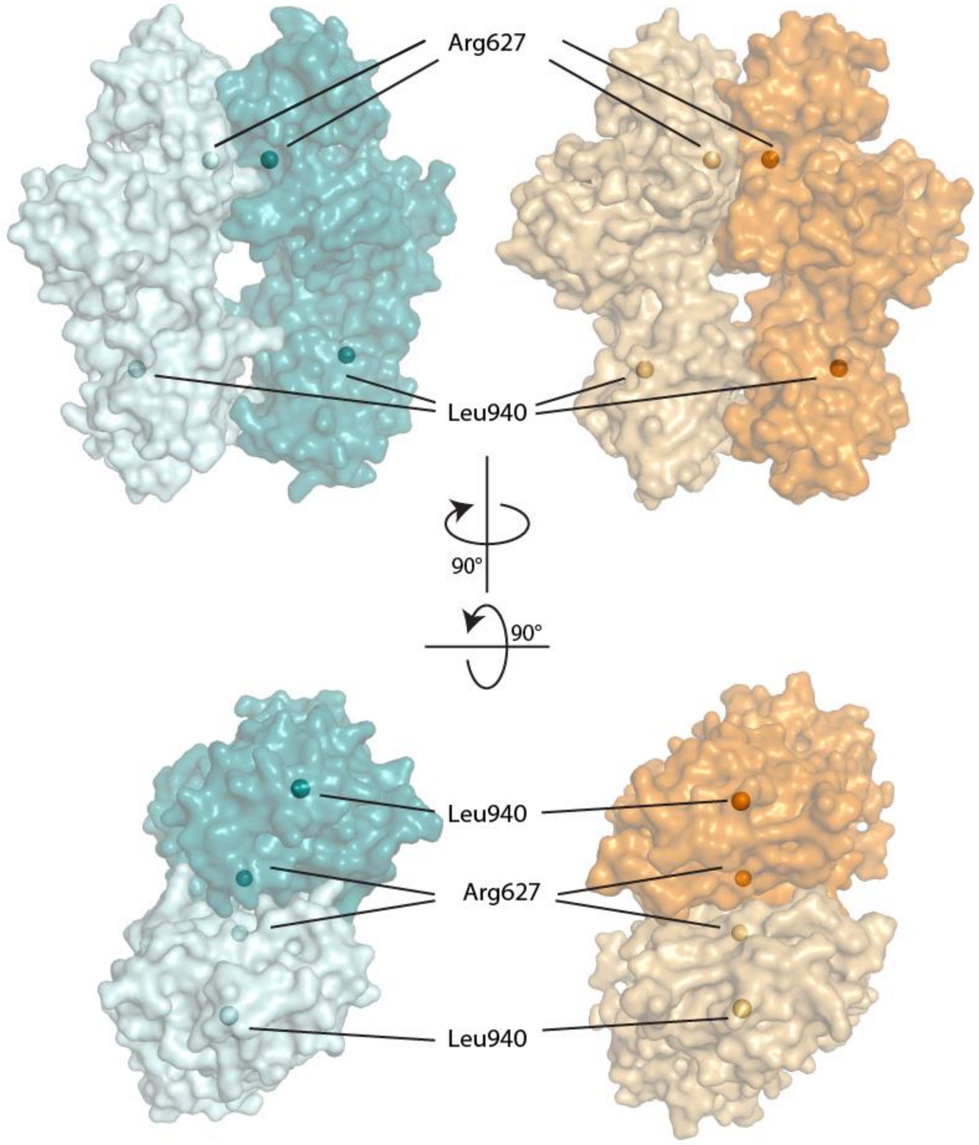

Figure S2: hIRE1 and yIRE1 protomers are twisted

Orthogonal views of the apo-hIRE1 (blue) and phos-yIRE1 (orange) are shown in combination with Figure 5D. Chains colored with different intensity. Specific conserved residues are indicated by spheres; Arg617 in the N-lobe interface and Leu940 within the hydrophobic core of the RNase domain (hIRE1 numbering).

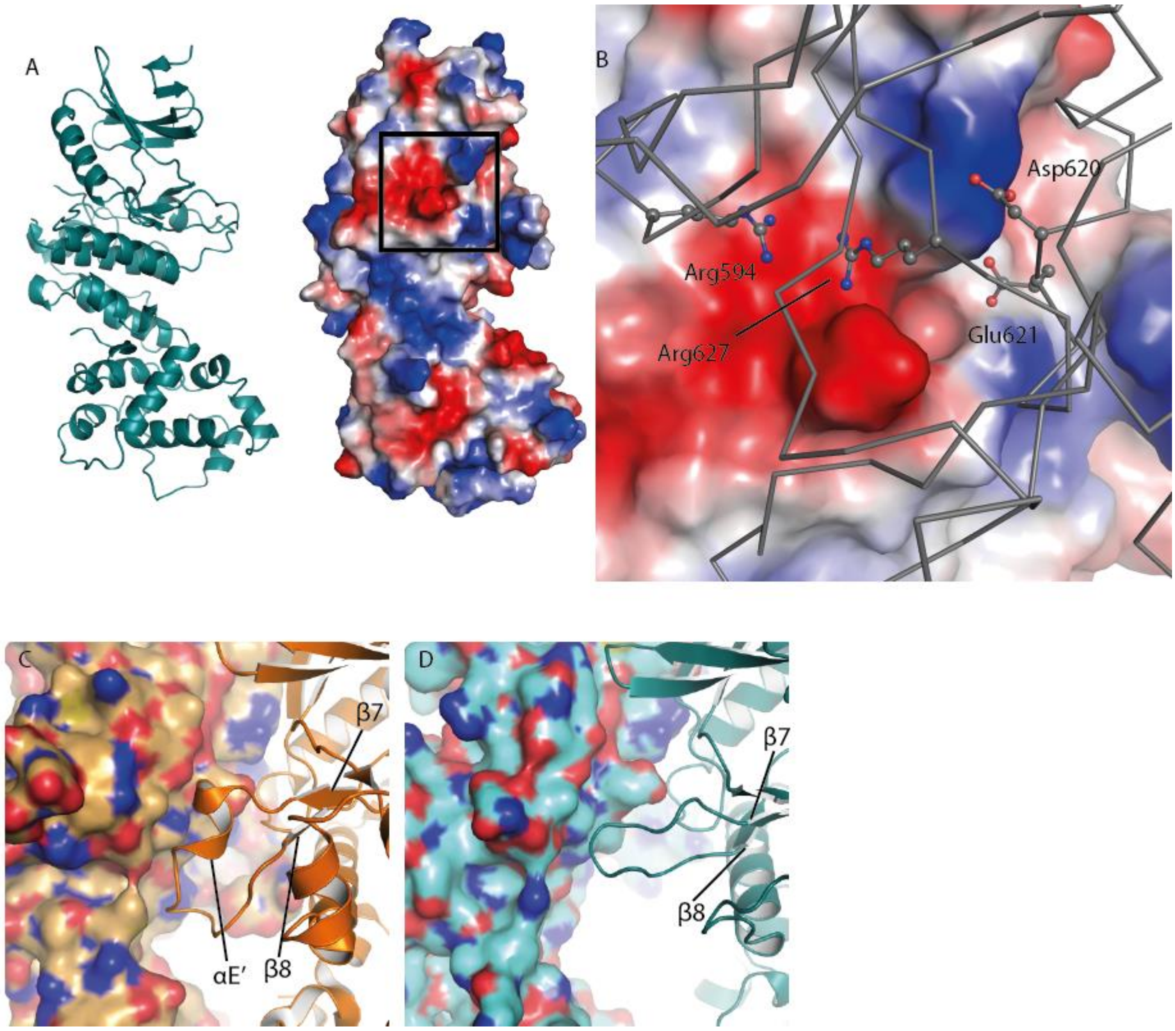

Figure S3: IRE1 dimer interactions within the kinase domain.

(A) Cartoon (left) and surface representation (right) of a monomer of apo-hIRE1. Surface view colored by vacuum electrostatics generated in PyMOL [44]. Blue indicates basic regions while red indicates acidic regions. Box in surface view is expanded in (B), which shows a surface representation of one apo-hIRE1 monomer colored by vacuum electrostatics. The second monomer is shown as Cα trace. Selected side chains are shown as ball-and-stick representation. Basic residues on one monomer interact with an acidic region on the partnering protein. (C) yIRE1 dimerisation interface in the vicinity of the αE′ helix, which lies between β7 and β8 in the C-lobe. One protomer is shown as a surface, the other in cartoon representation, carbon atoms are colored orange, oxygen atoms are colored red and nitrogen atoms are colored blue. (D) hIRE1 dimerisation interface in the region equivalent to that of the yIRE1 αE′ helix. hIRE1 has a loop region between β7 and β8 that is 8aa shorter than its equivalent in yIRE1 and which does not form an α-helix. hIRE1 carbon atoms are colored orange, oxygen atoms are colored red and nitrogen atoms are colored blue.

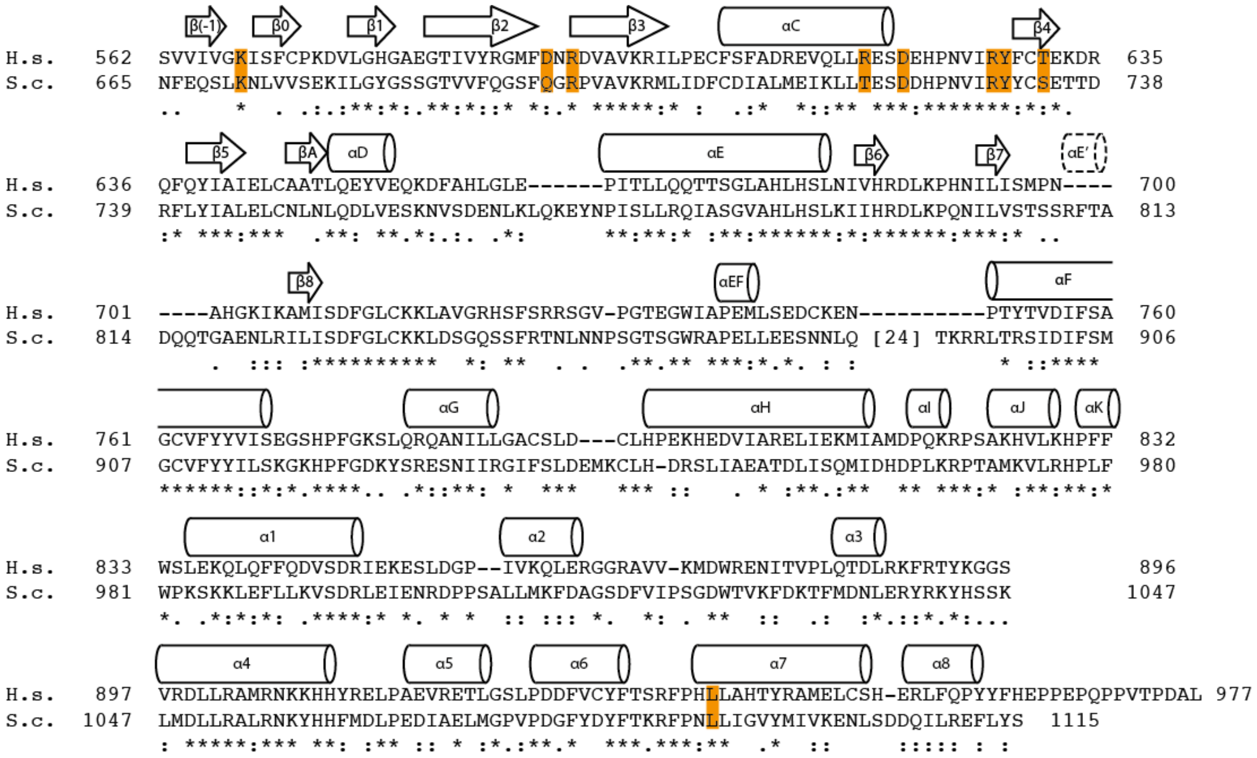

Figure S4: Sequence alignment of human and yeast IRE1.

Sequence alignment of hIRE1 and yIRE1 (yIRE1, S.c. UNIPROT: P32361; hIRE1, H.s. UNIPROT: O75460). Secondary structure elements are represented by arrow and cylinders (β-strands and α-helices respectively). The additional αE′ in yIRE1 is shown by a dashed cylinder. Residues involved in the N-lobe dimer interface and Leu940 (within the core of the RNase domain) are highlighted.

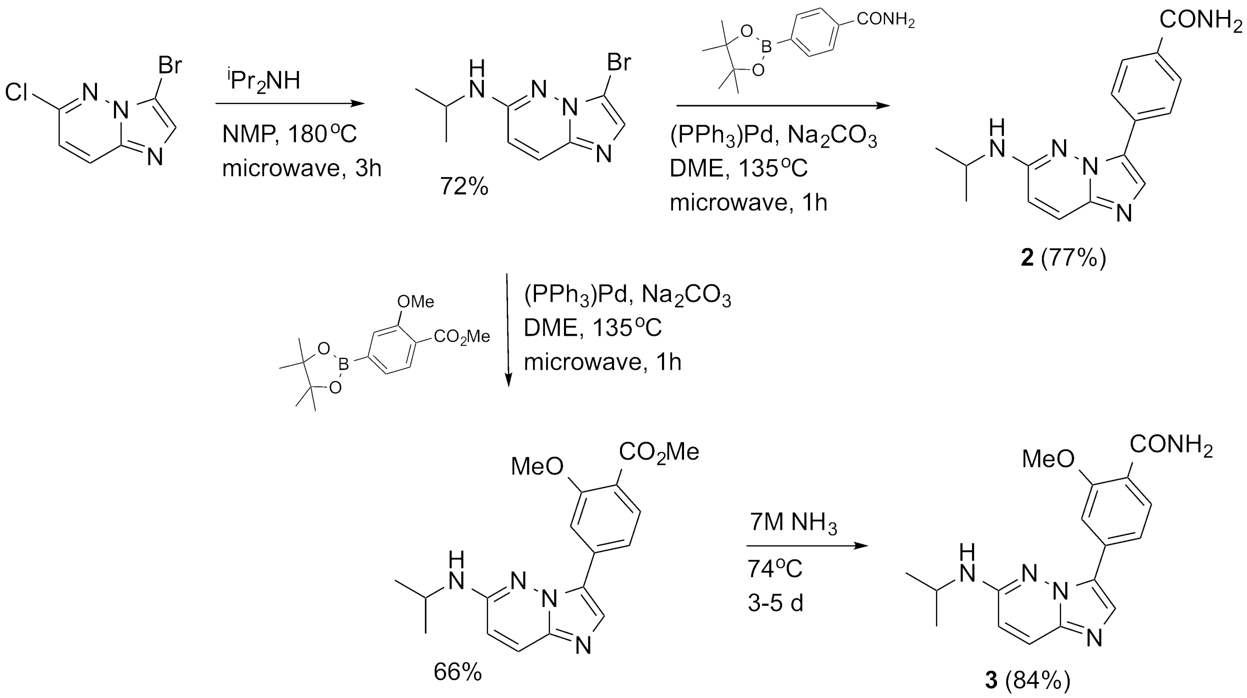

Figure S5: The chemical synthesis of 4-(imidazo[1,2-b]pyridazin-3-yl)benzamide inhibitors of hIRE1α.

A DELFIA medium-throughput screen for inhibitors of hIRE1 autophosphorylation was established and applied to a focused library of kinase inhibitor scaffolds, from which several structural types were identified as hIRE1 kinase inhibitors [29]. Among these were a series of 4-(imidazo[1,2-*b*]pyridazin-3-yl)benzamides exemplified by compound **1** (Figure 7A). Medicinal chemistry to explore the structure-activity relationship for inhibition of hIRE1 autophosphorylation led to the simpler scaffold **2** with enhanced hIRE1 kinase inhibitory activity. Exploration of the series was rapidly carried out using microwave-assisted reactions to assemble the inhibitors from the 3-bromo-6-chloroimidazo[1,2-*b*]pyridazine starting material.
